# Exploring 2-Tetradecanoylimino-3-aryl-4-methyl-1,3-thiazolines Derivatives as Alkaline Phosphatase Inhibitors: Biochemical Evaluation and Computational Analysis

**DOI:** 10.3390/molecules27196766

**Published:** 2022-10-10

**Authors:** Aftab Ahmed, Sajid-ur Rehman, Syeda Abida Ejaz, Aamer Saeed, Rabail Ujan, Pervaiz Ali Channar, Khalida Mahar, Reshma Sahito, Sarah M. Albogami, Qamar Abbas, Mohammed Alorabi, Michel De Waard, Gaber El-Saber Batiha

**Affiliations:** 1 Department of Pharmaceutical Chemistry, Faculty of Pharmacy, The Islamia University of Bahawalpur, Bahawalpur 63100, Pakistan; 2 Department of Chemistry, Quaid-I-Azam University, Islamabad 45320, Pakistan; 3 Dr. M. A. Kazi Institute of Chemistry, University of Sindh, Jamshoro 66020, Pakistan; 4 Department of Basic Sciences, Mathematics and Humanities, Dawood University of Engineering and Technology, Karachi 74800, Pakistan; 5 Institute of Chemistry, Shah Abdul Latif University, Khairpur 66020, Pakistan; 6 Department of Zoology, University of Sindh, Jamshoro 75500, Pakistan; 7 Department of Biotechnology, College of Science, Taif University, P.O. Box 11099, Taif 21944, Saudi Arabia; 8 Department of Biology, College of Science, University of Bahrain, Sakhir 32038, Bahrain; 9 Smartox Biotechnology, 6 Rue des Platanes, 38120 Saint-Egrève, France; 10 L’institut du Thorax, INSERM, CNRS, Université de Nantes, 44007 Nantes, France; 11 LabEx «Ion Channels, Science & Therapeutics», Université de Nice Sophia-Antipolis, 06560 Valbonne, France; 12 Department of Pharmacology and Therapeutics, Faculty of Veterinary Medicine, Damanhour University, Damanhour City 22511, Egypt

**Keywords:** 1-aroyl-3-arylthioureas, alkaline phosphatase, density functional theory, molecular docking

## Abstract

The current study focused on the laboratory approach in conjunction with computational methods for the synthesis and bioactivity assessment of unique 2-tetradecanoylimino-3-aryl-4-methyl-1,3-thiazolines (**2a**–**2k**). Processes included cyclizing 1-aroyl-3-arylthioureas with propan-2-one in the presence of trimethylamine and bromine. By using spectroscopic techniques and elemental analyses, structures were elucidated. To assess the electronic properties, density functional theory (DFT) calculations were made, while binding interactions of synthesized derivatives were studied by the molecular docking tool. Promising results were found during the evaluation of bioactivity of synthesized compounds against alkaline phosphatase. The drug likeliness score, an indicator used for any chemical entity posing as a drug, was within acceptable limits. The data suggested that most of the derivatives were potent inhibitors of alkaline phosphatase, which in turn may act as lead molecules to synthesize derivatives having desired pharmacological profiles for the treatment of specific diseases associated with abnormal levels of ALPs.

## 1. Introduction

Alkaline phosphatases (ALPs) are the isozymes concentrated in the outer layer of the cell membrane. The clinical relevance of ALPs in various diseases, especially hepatic disorders, makes them an important area of research among medicinal chemists. Elevated ALP levels are considered in the diagnostic procedure of a wide variety of bile duct and liver-related morbidities, while abnormally low levels are presented in Wilson disease [1]. Alkaline phosphatase, a subclass of the superfamily of hydrolase enzymes, acts as a catalyst in the hydrolysis of various phosphates, pyrophosphate, sulphate, and sulphonate esters. Four alkaline phosphatase isozymes are expressed in the human body, including three tissue-specific ALPs and one non-tissue-specific ALP (TNAP). Tissue-specific ALPs are localized in placenta, germ cells, and intestine while tissue-nonspecific ALPs are concentrated in bone, kidney, liver, and the central nervous system [2,3,4,5,6,7]. Human serum also contains ALPs and has been used in the diagnosis of liver disorders, bone diseases, and multiple carcinomas [7,8,9]. Human intestinal ALPs (h-IAPs) are located at the intestinal brush border. Important roles of h-IAPs include –HCO_3_ secretion to maintain pH, intestinal lipopolysaccharides detoxification, and the regulation of lipid absorption [10,11]. Decreased circulating level of ALPs results in inflammation due to weakened effects of endotoxin detoxification [12], while upregulation occurs in inflammatory bowel disease. Limited numbers of h-IAPs are known, owing to the fact that the structural similarity among TNAP and IAP is higher [13]. Due to wider implications of alkaline phosphatase, the search for a selective and potent inhibitor of ALPs is of greatest importance. A literature review indicated the efficient potential of thiazole derivatives as alkaline phosphatase inhibitors [14,15].

**Figure 1:**
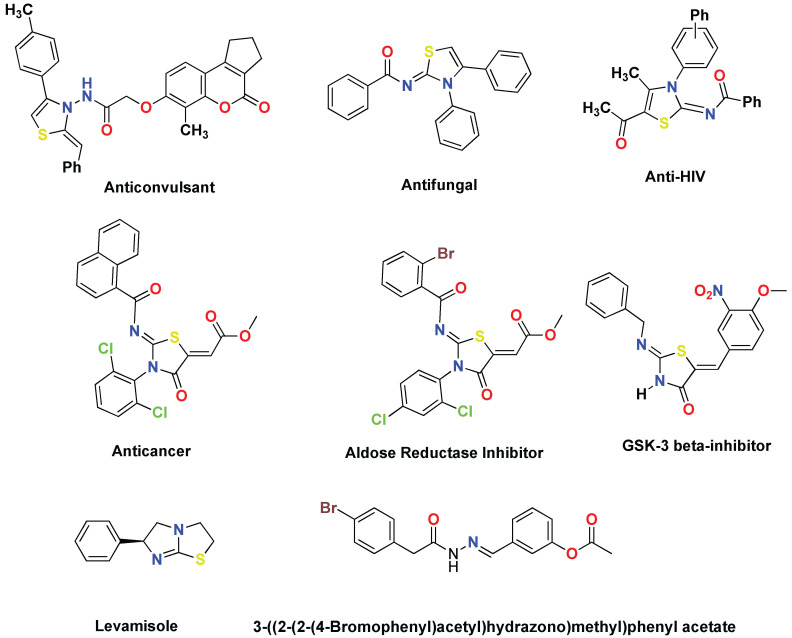
Chemical structures of pharmacologically important compounds [33,34,35,36].

The pharmacological effects of thiazole compounds include anticonvulsant, antihistaminic, antimicrobial, hypnotic, anti-inflammatory, and antihypertensive [16,17,18,19,20,21]. According to reports, rhodanine’s thiazolidinone derivatives are effective against germs, viruses, pests, inflammation, and diabetes [22,23,24,25]. The selectivity of hepatitis C virus NS3 protease’s selectivity was improved by the formation of an arylalkylidene rhodanine library and subsequent changes in rhodanine side chains [25]. Promising antitumor activities of bis-thiazole derivatives have been found in human cell lines [26]. A literature review indicated that the use of thiazoines as an acaricidal and an insecticidal is well established. In addition to their activity as plant growth regulation, thiazoines have diagnostic applications in human MPO (myeloperoxidase) blood tests [27,28,29,30]. Considerable antifungal activity of 2-phenylimino-1,3-thiazoline-4-acetanilides have been demonstrated against *Pyricularia oryzae* [31]. The condensation products of 9-chloro-2,4-(un)substituted acridines with 3-aryl-4-phenyl-2-imino-4-thiazolines showed intriguing analgesic and anti-inflammatory properties [32]. Heterocyclic compounds with the thiazoline moiety have several pharmacological applications in the drug industry. These compounds exhibit anticonvulsant [33], antifungal [34], and anti-HIV [35] activity. An anticancer, a GSK-3 beta-inhibitor, and known alkaline phosphatase inhibitors, i.e., levamisole and 3-((2-(2-(4-bromophenyl)acetyl)hydrazono)methyl)phenyl acetate [36], are shown in Figure 1. In light of the unique structural features and important biological activities, 2-iminothiazoline derivatives have drawn considerable attention of synthetic chemists for their preparation.

In the search for potent, selective, and drug-like molecules of intestinal alkaline phosphatase inhibitor intended to be used in human populations, the synthesis of 2-tetradecanoylimino-3-aryl-4-methyl-1,3-thiazolines derivatives was carried out.

Other strategies for the synthesis of 2-iminothiazoles include alkylation of 2-aminothiazoles [20], the reaction of α-bromoketamines with potassium thiocyanate [37], the reaction of α-chloroketones with thiosemi-carbazide [38], and the reaction of ketones with bisbenzyl formamidine disulfide or N-alkyl rhodanamines [39]. Currently, 2-imino-1,3-thiazoline synthesis has been reported to have been carried out by the ring transformation of 1-arylmethyl-2-(thiocyanomethyl) aziridines [40]. 

In the present study, new 2-tetradecanoylimino-3-aryl-4-methyl-1,3-thiazolines were synthesized by considering the advantage of avoidance of unwanted side products and overall high yields. With the acetone cyclization of corresponding 1-tetradecanoyl-3-arylthiourea taking place, the reaction takes place in the presence of bromine and triethyl amine.

To gain insights into geometric and electronic behaviors, density functional theory (DFT) studies were performed. In vitro free radical scavenging activity and alkaline phosphatase inhibition activity were determined. To study the binding interactions of synthesized derivatives with alkaline phosphatase, molecular docking was performed. Drug likeliness, an important characteristic of any chemical entity to be purposed as a medicinal agent, was calculated using an online tool. 

## 2. Experimental

### 2.1. Chemistry

*2-Tetradecanoylimino-3-aryl-4-methyl-1,3-thiazolines synthesis (**2a–2k**)*

**Figure 2:**
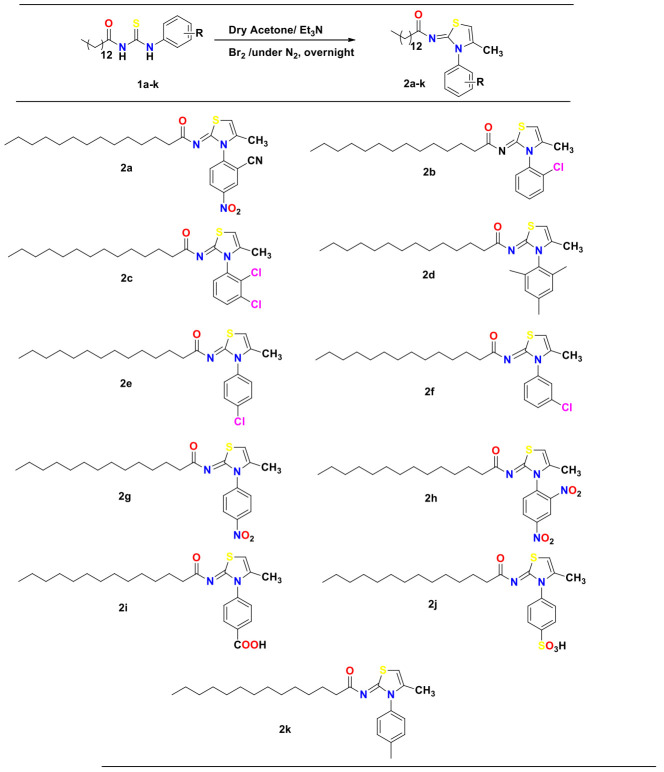
2-Tetradecanoylimino-3-aryl-4-methyl-1,3-thiazolines derivatives synthesis (**2a**–**2k**).

Under a nitrogen gas atmosphere, bromine solution (0.1 mL) in dry acetone (10 mL) was added dropwise to a 1-tetradecanoyl-3-arylthiourea (0.5 g) solution under stirring in 20 mL dry acetone that contained 0.3 mL triethylamine. The next step was the overnight stirring of the solution at room temperature after the final reactant was added. To track the reaction’s progress, the thin layer chromatographic technique was employed. To remove crude solids, the reaction mixture was concentrated and filtered. By recrystallizing with ethanol, derivatives of 2-tetradecanoylimino-3-aryl-4-methyl-1,3-thiazoline were collected. The scheme of synthesis in given in Figure 2. 

### 2.2. Biological Evaluation

#### 2.2.1. Alkaline Phosphatase Assay

As already described by Jamshed et al. in 2011, the spectrophotometric technique was used for the assessment of calf intestinal alkaline phosphatase (CIALP) activity [41]. The reaction mixture included 50 mM Tris-HCl buffer with 0.1 mM ZnCl_2_ and 5 mM MgCl_2_ (pH 9.5) and test substance (0.1 mM with final DMSO 1% (*v*/*v*). Then 0.025 U/mL CIALP was added to the pre-incubated mixture. Mixing was conducted for 10 min. The reaction mixture was then added with 10 µL of the substrate, having 0.5 mM p-NPP (para nitrophenylphosphate disodium salt). It was once more incubated for 30 min at 37 °C. A change in absorbance of the released p-nitrophenolate was observed at 405 nm with a 96-well microplate reader (OPTI MAX, Milton Freewater, OR, USA). Triplicate independent repetition of experiments was done. As a reference inhibitor of calf ALP, KH_2_PO_4_ was used.

#### 2.2.2. Free Radical Scavenging Assay

By modifying the previously described method [42] the 2,2-diphenyl-1 picrylhydrazyl (DPPH) assay was used to assess the radical scavenging activity. The reaction mixture contained 20 µL of test compounds at progressively higher concentrations, 100 µL of DPPH (150 µM), and 200 µL of methanol to adjust the volume in each well. At room temperature, the reaction mixture was let to sit for another 30 min. The reference inhibitor utilized was ascorbic acid (also known as vitamin C). Measurements were taken using an OptiMax tunable microplate reader for the wavelength of 517 nm comparing reaction rates and calculating the % inhibition brought on by the presence of the tested inhibitors. The independent repetition of experiments was done in a triplicate manner.

### 2.3. Computational Studies

#### 2.3.1. Density Functional Theory (DFT) Calculations

Density functional theory studies are a computational quantum mechanical modelling technique used for the prediction of the electronic properties of chemical compounds. For quantum mechanical calculations, frontier molecular orbitals (HOMO, LUMO), the molecular electrostatic map, and electron density were measured. DFT studies were conducted using Gaussian 16 on a workstation having an AMD Ryzen 9 5950x @ 16 core processor with 64 GB of installed RAM memory (Santa Clara, CA, USA). For representing the quantum mechanics region, the density functional theory (DFT) method is critical; all calculations were done using the B3LYP/6-311G basis set. Visualization of calculations were performed using Gauss view 6 [43,44].

#### 2.3.2. Molecular Docking Studies

The Protein Data Bank (PDB) https://www.rcsb.org/ was used to retrieve the three-dimensional (3D) structure of human placental alkaline phosphatase, having the PDB ID: 1ZED, accessed on 18 September 2022. Utilizing the Discovery Studio Visualizer 2021 software, the chosen protein structure was minimized.

The structures of all synthesized derivatives and the reference compound p-nitrophenyl-phosphonate were drawn using ChemDraw 12.0 followed by energy minimization by using Chem3D Pro 12.0. In addition to an active pocket, alkaline phosphatases have peripheral allosteric binding sites, which play roles in enzyme regulation. Keeping in mind the enzyme structural studies, dimensions of p-nitrophenyl-phosphonate (PNP) were used for molecular docking studies. The reason for the selection of p-nitrophenyl-phosphonate as the reference ligand is its binding capability with placental alkaline phosphatase [45]. 

The dimensions of PNP 902, presented as a ligand of interest, were taken, i.e., x = +57.430500, y = 28.435571, and z = −2.404500 with size values of X = 40, Y= 40, and Z= 40. The value of 8 was taken as the default exhaustiveness. Synthesized ligands (**2a**–**2k**) were docked using AutoDock Vina [46] against alkaline phosphatase. The predicted docked complexes were analyzed using the values of binding energy (Kcal/mol). Ligand–protein interactions were visualized in three dimensions (3D) and two dimensions (2D) using Discovery Studio 21 [47]. 

#### 2.3.3. Chemo-Informatics Analysis of Ligands 

The ACD/ChemSketch tool was used to sketch the synthesized chemical structures (**2a**–**2k**). The molecular weight (g/mol), density, hydrogen bond acceptors (HBA), hydrogen bond donors (HBD), polarizability, logP, molecular volume (A3), molar refractivity, and drug likeness score were among the basic chemo-informatics features assessed using Molinspiration (http://www.molinspiration.com/) and Molsoft (http://www.molsoft.com/) online tools (accessed on 18 September 2022).

## 3. Results and Discussion

### 3.1. Chemistry

In accordance with reported method [48], a series of 2-tetradecanoylimino-3-aryl-4-methyl-1,3-thiazolines derivatives was synthesized by reacting 1-tetradecanoyl-3-arylthioureas (**1a**–**1k**) with bromine in dry acetone in the presence of triethyl amine to afford the cyclized 1,3-thiazolines derivatives (**2a**–**2k**), as delineated in Figure 2. The synthesized compounds were characterized by FT-IR and NMR spectroscopy. FT-IR spectra of synthesized compounds exhibited absorptions for the C–H aromatic at 3020 cm^−1^, C–H thiazoline at 2917 cm^−1^, and C=O at 1642 cm^−1^. The 1H NMR spectra of compound (**2a**–**2k**) contained the characteristic one proton singlet of the thiazoline ring at δ 6.40 ppm. The alkyl protons gave rise to signals at δ 2.50–0.86, whereas the aromatic protons appeared in the range δ 7.53–7.21 ppm. In 13C NMR spectra, the signal for carbonyl carbon appeared at δ 182.1 ppm, and for imine carbon of the thiazoline ring, it was observed at δ 169.4 ppm. The aromatic carbons were observed in the range of δ 138.1–112.4 and aliphatic carbons in the region of δ 39.4–14.2 ppm. Characterization data are included in the Supplementary File, and IR and NMR spectra of important synthesized derivative are given in the Supplementary File, Figures S1–S8. 

### 3.2. Biological Evaluation

#### 3.2.1. Alkaline Phosphatase Assay

All the synthesized compounds (**2a**–**2k**) were evaluated for their inhibition potential to calf intestinal alkaline phosphatase (CIAP). The standard used was KH_2_PO_4_, and the results of evaluation as half maximal inhibitory concentration (IC_50_) are summarized in Table 1.

#### 3.2.2. Structure–Activity Relationship

Remarkably, most of the synthesized compounds exhibited many-fold inhibition potential towards alkaline phosphatase compared to the standard. Compounds **2a**, **2k,** and **2c** showed better and more efficient inhibition potential, as represented by their respective IC_50_ values. Compound **2h,** with two nitro atoms at the 2- and 4-positions of the phenyl ring, was found to be the least potent due to their electron withdrawing nature. Compound **2k**, containing a 4-methyl group, was found to be moderately potent. Compounds with cyano and carboxylic and sulfonic acid substitutions at the 4-position showed various degrees of inhibition. The derivative **2f**, having a chlorine atom at the 3-position, was found to be the most potent among the series, which may be attributed to the electron-withdrawing inductive effect of the chlorine atom, which perturbs the electron density and possibly results in the enhancement of alkaline phosphatase activity. In general, compounds with an electron donating nature showed better inhibition of CIAP as compared to those with an electron withdrawing nature.

#### 3.2.3. Free Radical Scavenging Activity

**Figure 3:**
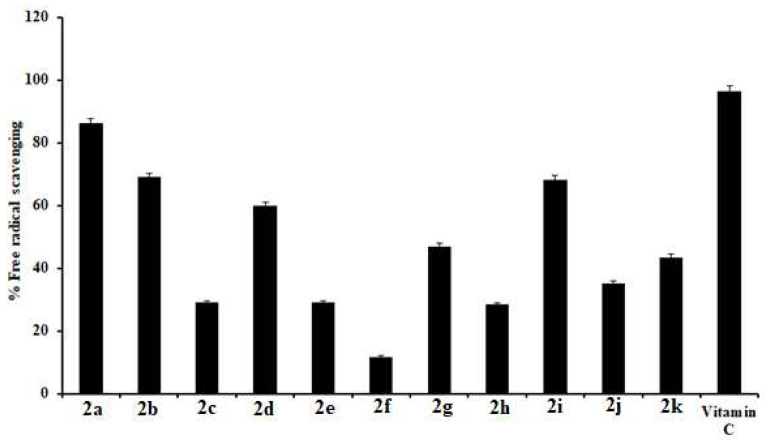
Graphical representation of percentage free radical scavenging activity. Values are given as mean ± SEM. Concentrations of tested compounds = 100 µg/mL.

Radical scavenging activities of compounds (**2a**–**2k**) were compared, with vitamin C used as a reference. Compound **2a** exhibited maximum potency, comparable to the standard ascorbic acid. Compounds **2a**, **2b**, **2d,** and **2i** also showed significant radical scavenging potency. The remaining compounds were quite inactive. % age free radical scavenging activity is graphically represented in Figure 3. 

### 3.3. Computational Studies

#### 3.3.1. Density Functional Theory (DFTs) Calculations

**Figure 4:**
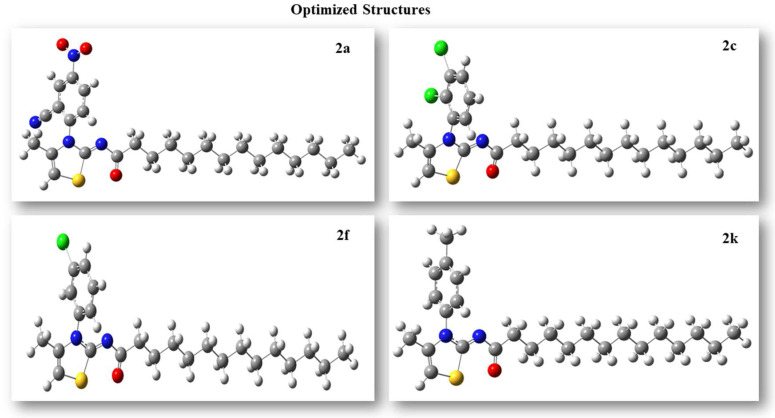
Optimized structures of compounds **2f, 2a, 2k,** and **2c**.

DFT calculations of 2-tetradecanoylimino-3-aryl-4-methyl-1,3-thiazolines derivatives (**2a**–**2k**) including optimization, global reactivity descriptors, and FMOs analysis were made using the B3LYP/6-311G basis set. All derivatives were optimized to the steepest energy gradient. Optimized structures of the most potent compounds are given in Figure 4, while other structures are given in the supplementary information (Figure S9 from Supplementary Materials).

Optimization energy, polarizability, and dipole moment play significant roles in determining the chemical reactivity of a compound. The analysis of the HOMO and LUMO energies facilitated the understanding of the various molecular properties as well as electrical and optical properties of the molecule. Moreover, the highest occupied molecular orbital (HOMO) and the lowest unoccupied molecular orbital (LUMO) energy gap also depict the chemical reactivity of compounds. Compounds having small HOMO and LUMO energy gaps are referred to as reactive molecules, whereas large energy gaps correspond to stability and less reactivity.

Table 2 shows the optimization energies, dipole moments, polarizability, HOMO and LUMO energies, and their energy gaps. Compound **2d**, which has the highest energy gap (ΔEgap = 0.182) tended to be a stable molecule. Similarly, compound **2h** had the lowest energy gap (ΔEgap = 0.092), justifying its highest reactivity. The molecule with the highest HOMO energy, **2a** (EHOMO = −0.226), turned out to be the best electron donor, while compound **2k**, which had the lowest ELUMO of −0.024 was shown to be the best electron acceptor.

**Figure 5:**
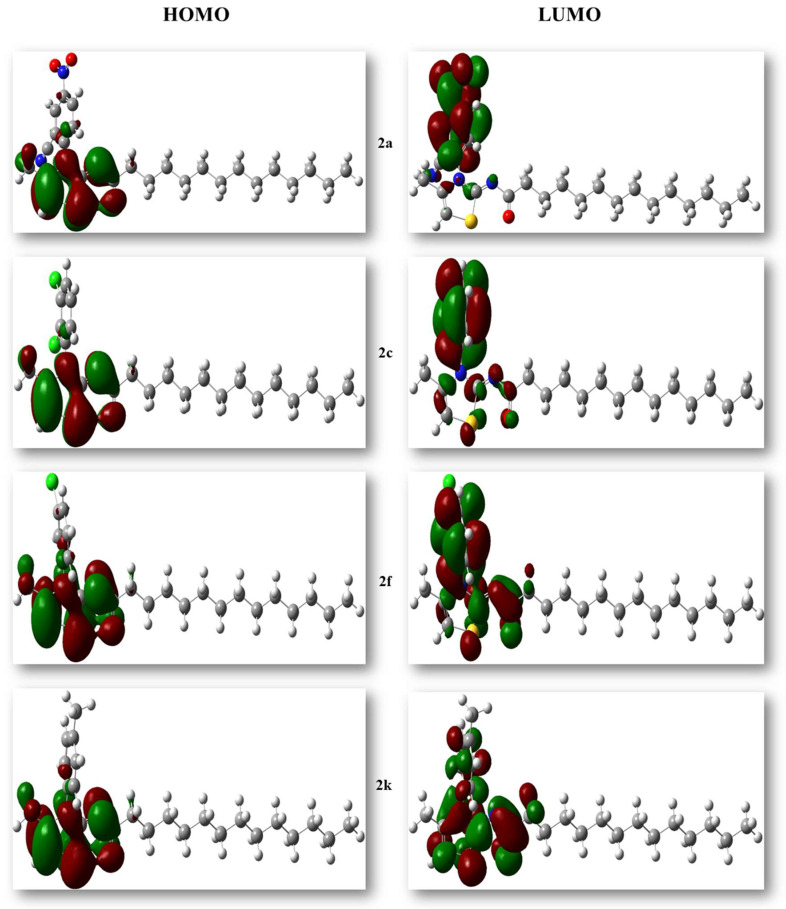
HOMO–LUMO orbitals (**2a**, **2c**, **2f**, **2k**). Red, yellow, and blue atoms indicate O, S, and N atoms.

HOMO and LUMO orbitals of the most potent compounds are visualized in Figure 5, while HOMO and LUMO orbitals of the remaining compounds are shown in Figure S10 in the Supplementary Materials. 

*Global chemical reactivity descriptors*

By using the highest occupied molecular orbital (HOMO) and lowest unoccupied molecular orbital (LUMO) energy values, we evaluated the following parameters by using their respective formulas [49]. Global reactivity descriptor’s values are given in Table 3.

Compound **2h** had the highest chemical potential and electronegativity. Similarly, compound **2d** was the harder compound, whereas compound **2h** was the softer compound. Compound **2a** had the highest electrophilicity index. 

#### 3.3.2. Molecular Docking Studies

##### Molecular Docking Analysis

Synthesized compounds (**2a**–**2k**) were docked against alkaline phosphatase for the prediction of the best conformational position in the active pocket of the target protein. On the basis of energy values (Kcal/mol) and hydrogen/hydrophobic interactions patterns, docked complexes were analyzed. Binding energy values of synthesized compounds and reference ligand are given in Table 4. Due to the fact that all synthesized compounds had the same basic chemical nucleus, significant energy difference are not shown. 

*Binding Interactions*

**Figure 6:**
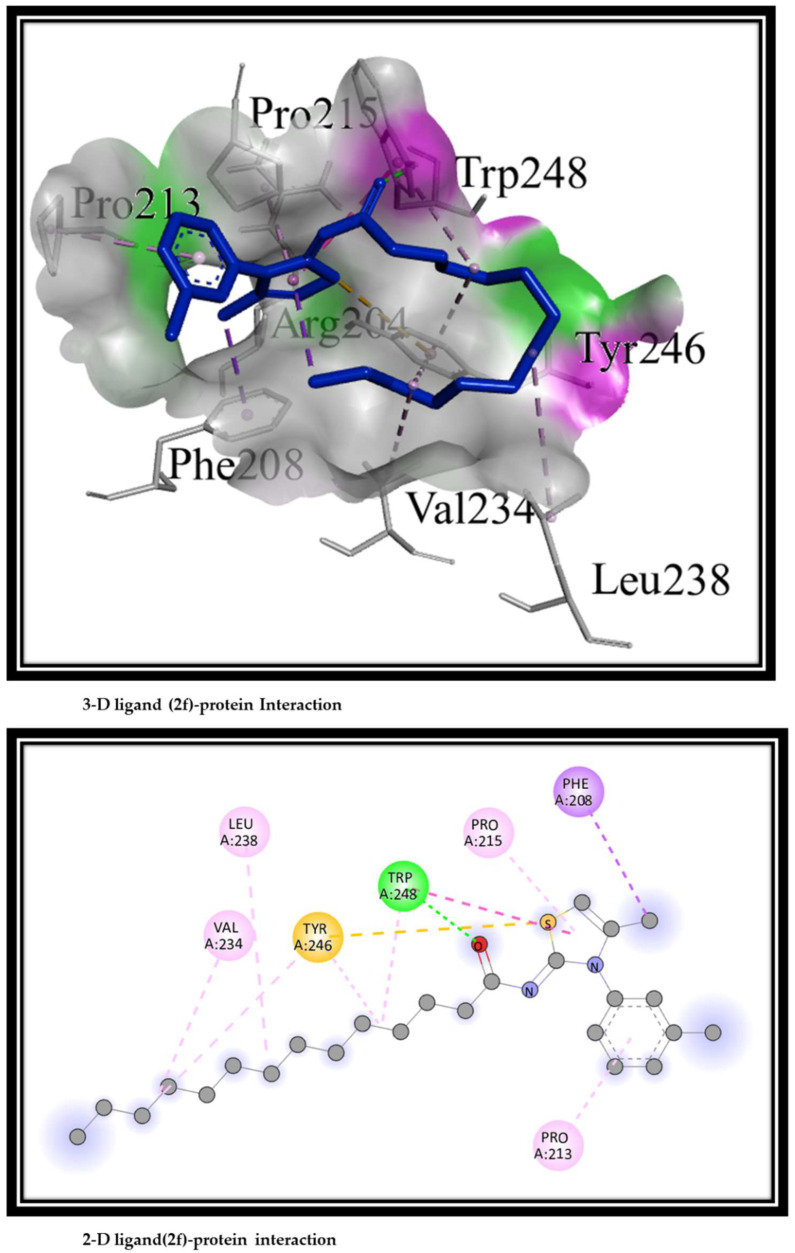
The 2D and 3D protein ligand (**2f**) interactions.

The 3D and 2D interactions of the most potent ligand, **2f**, are shown in Figure 6.

Amino acid residues present in the active pocket that are responsible for binding interactions are: PHE208, THR212, PRO213, ASP214, PRO215, ASN232, VAL234, GLN235, LEU238, TYR246, VAL247, and TRP248.

Figure 6 shows that all ligands were contained in the active pocket of alkaline phosphatase. Amino acid residue TRP248 was involved in three types of interactions, i.e., conventional hydrogen bond with carbonyl oxygen, Pi–Pi T-shaped interaction with a thiazole ring, and alkyl interaction with an aliphatic side chain. TYR246 contributed in the formation of the Pi sulphur bond with a sulphur atom of the thiazole ring and two alkyl interactions with the aliphatic side chain. Amino acid PHE208 formed Pi Sigma interactions with the methyl group attached with thiazole ring.VAL234 and LEU238 were involved in alkyl interactions with the side chain, while PRO213 and PRO215 took part in Pi alkyl interactions with phenyl ring and thiazole rings, respectively. In vitro studies and molecular docking studies suggested the potency of derivative **2f** as an alkaline phosphatase inhibitor.

The 2D and 3D interactions of human placental ALP with reference ligand para nitrophenyl phosphonate are given in Supplementary File Figure S11 from Supplementary Materials.

### 3.4. Chemo-Informatics Analysis

The expected attributes including polar surface area (PSA), density, molar volume, logP, molecular weight, and RO5 were predicted to justify their drug-like behavior [50]. According to research results, for the assessment of drug absorption in the drug development process, calculation of PSA is crucial [51]. Lipophilicity and molar refractivity play important roles in binding to receptors, bioavailability, and cellular absorption. According to earlier research, the usual ranges of molecular weight of 160 to 480 g/mol, molar refractivity of 40 to 130 cm^3^ and polar surface area (PSA) of 89 are considered optimal [52]. For the total number of atoms in the drug-like molecule, the permitted range is between 20 and 70 [53]. The results shown in Table 5 indicate that all the predicted chemo-informatic properties of synthesized compounds lied within the standard ranges except logP. Furthermore, compounds **2a**–**2k** were validated by Lipinski’s rule. Our results justified that, except for logP values, all other parameters were followed, so the Lipinski rule was fulfilled. Lipinski violation should be employed for the compounds with poor absorption. However, large number of drug molecules are present that violate RO5 [54]. 

## 4. Conclusions

In the present study, 11 synthetic derivatives of 2-tetradecanoylimino-3-aryl-4-methyl-1,3-thiazolines were synthesized and characterized. Lab data of the alkaline phosphatase inhibition assay revealed the higher potency of **2f**, **2a**, **2e** and **2k**. IC_50_ values were correlated with molecular docking studies, which verified the lab results. Important information about electronic properties and global reactivity descriptors were obtained through DFT calculations. Finally, chemo-informatic analyses were made to justify their behavior as drug-like molecules. Results were found within the acceptable limits. Thus, these compounds are an important addition among ALPIs. However further studies are required for their use as medicinal agents for the treatment of diseases linked with alkaline phosphatase abnormalities.

## Figures and Tables

**Table 1:** Alkaline phosphatase inhibitory activity of compounds **2a**–**2k**.

Compound	Alkaline Phosphatase IC_50_ (µM)
**2a**	0.019 ± 0.001
**2b**	0.193 ± 0.004
**2c**	0.052 ± 0.011
**2d**	0.113 ± 0.021
**2e**	0.086 ± 0.011
**2f**	0.015 ± 0.011
**2g**	0.211 ± 0.003
**2h**	0.342 ± 0.011
**2i**	0.292 ± 0.015
**2j**	0.136 ± 0.002
**2k**	0.032 ± 0.001
**KH_2_PO_4_**	4.28 ± 0.311

Values are presented as mean ± SEM.

**Table 2:** Electronic properties of synthesized compounds (**2a***–***2k**).

Comp.	Optimization Energy	Dipole Moment	Polarizability (α)	HOMO (eV)	LUMO (eV)	LUMO–HOMO (ΔeV)
**2a**	−1806.171	3.755	310.336	−0.226	−0.123	0.103
**2b**	−1968.498	5.928	285.312	−0.209	−0.0365	0.173
**2c**	−2425.884	5.285	296.332	−0.214	−0.048	0.167
**2d**	−1628.423	5.399	309.696	−0.207	−0.025	0.182
**2e**	−1968.500	3.970	288.829	−0.2131	−0.038	0.175
**2f**	−1968.500	4.385	288.499	−0.2134	−0.040	0.174
**2g**	−1714.446	3.681	298.769	−0.2214	−0.107	0.115
**2h**	−1917.773	4.230	309.403	−0.223	−0.132	0.092
**2i**	−1699.835	4.326	299.960	−0.185	−0.042	0.144
**2j**	−2132.772	4.777	312.171	−0.195	−0.047	0.148
**2k**	−1550.211	6.024	289.314	−0.205	−0.024	0.181

**Table 3:** Global reactivity descriptors.

Comp.	Chemical Potential µ	Softness S	Hardness ƞ	Electrophilicity Index ω	Electronegativity X
**2a**	−0.175	9.720	0.051	0.296	0.175
**2b**	−0.123	5.782	0.086	0.087	0.123
**2c**	−0.131	6.002	0.083	0.103	0.131
**2d**	−0.115	5.484	0.091	0.073	0.115
**2e**	−0.125	5.701	0.088	0.090	0.125
**2f**	−0.127	5.764	0.087	0.092	0.127
**2g**	−0.164	8.730	0.057	0.235	0.164
**2h**	−0.178	10.918	0.046	0.344	0.178
**2i**	−0.114	6.965	0.072	0.090	0.114
**2j**	−0.121	6.750	0.074	0.099	0.121
**2k**	−0.114	9.720	0.091	0.072	0.114

**Table 4:** The docking energy values of all synthetic derivatives.

Compound	Binding Energy (Kcal/mol)
**2a**	4.9
**2b**	5
**2c**	4.9
**2d**	4.7
**2e**	4.6
**2f**	5.3
**2g**	4.7
**2h**	4.7
**2i**	4.8
**2j**	4.4
**2k**	5.1
**Ref. (PNP)**	5.0

**Table 5:** Cheminformatics properties (**2a**–**2k**).

Comp.	MW	nHBA	nHBD	LogP	PSA (A2)	Volume (A3)	Drug Score
**2a**	470.24	6	0	7.05	79.81	513.39	−1.85
**2b**	434.22	3	0	8.12	24.49	470.26	−0.56
**2c**	468.18	3	0	8.71	24.49	485.93	−0.36
**2d**	442.30	3	0	8.49	24.19	516.74	−0.70
**2e**	434.22	3	0	8.24	24.79	471.18	−0.08
**2f**	434.21	3	0	8.20	24.60	470.18	−0.09
**2g**	445.24	5	0	7.25	63.05	479.62	−0.65
**2h**	490.22	7	0	6.86	100.71	506.39	−1.51
**2i**	444.24	5	1	7.23	53.20	487.09	−0.38
**2j**	480.21	6	1	7.60	67.20	496.89	−0.69
**2k**	414.27	3	0	7.92	24.79	474.93	−0.06

## Data Availability

Not applicable.

## Supplementary Materials

The following are available online at https://www.mdpi.com/article/10.3390/molecules27196766/s1.

## Sample Availability

Samples of the compounds are available from the authors.
