# Supplementary material for: Exploring 2-Tetradecanoylimino-3-aryl-4-methyl-1,3-thiazolines Derivatives as Alkaline Phosphatase Inhibitors: Biochemical Evaluation and Computational Analysis
Source: Molecules. 2022 Oct 10;27(19):6766. doi: 10.3390/molecules27196766 (PMC9572939; doi:10.3390/molecules27196766)
Supplement: Supplementary file 1 [file molecules-27-06766-s001.zip › molecules-1916221-supplementary.pdf]

molecules

MDPI

Supplementary Materials

Exploring 2-Tetradecanoylimino-3-aryl-4-methyl-1, 3-thiazolines derivatives as Alkaline Phosphatase Inhibitors: Biochemical Evaluation and Computational Analysis

Aftab Ahmed [1], Sajid-ur Rehman [2], Syeda Abida Ejaz [1,*], Aamer Saeed [2,*], Rabail Ujan [3], Pervaiz Ali Channar [2,4], Khalida Mahar [5], Reshma Sahito [6], Sarah M. Albogami [7], Qamar Abbas [8], Mohammed Alorabi [7], Michel De Waard [9,10,11] and Gaber El-Saber Batiha [12]

[1] Department of Pharmaceutical Chemistry, Faculty of Pharmacy, The Islamia University of Bahawalpur, Bahawalpur 63100, Pakistan
[2] Department of Chemistry, Quaid-I-Azam University, Islamabad 45320, Pakistan
[3] Dr. M. A. Kazi Institute of Chemistry, University of Sindh, Jamshoro, 66020, Pakistan
[4] Department of Basic Sciences, Mathematics and Humanities, Dawood University of Engineering and Technology, Karachi 74800, Pakistan
[5] Institute of Chemistry, Shah Abdul Latif University, Khairpur 66020, Pakistan
[6] Department of Zoology, University of Sindh, Jamshoro, Pakistan
[7] Department of Biotechnology, College of Science, Taif University, P.O. Box 11099, Taif 21944, Saudi Arabia
[8] Department of Biology, College of Science, University of Bahrain, Sakhir 32038, Bahrain
[9] Smartox Biotechnology, 6 rue des Platanes, 38120 Saint-Egrève, France
[10] L'institut du Thorax, INSERM, CNRS, Université de Nantes , 44007 Nantes, France
[11] LabEx «Ion Channels, Science & Therapeutics», Université de Nice Sophia-Antipolis, 06560 Valbonne, France
[12] Department of Pharmacology and Therapeutics, Faculty of Veterinary Medicine, Damanhour University, Damanhour City, Egypt
* Correspondence: abida.ejaz@iub.edu.pk or abidaejaz2010@gmail.com (S.A.E.); aamersaeed@yahoo.com (A.S.); Tel: +92-062-9250245 (S.A.E.); Fax: +92-062-9250245 (S.A.E.)

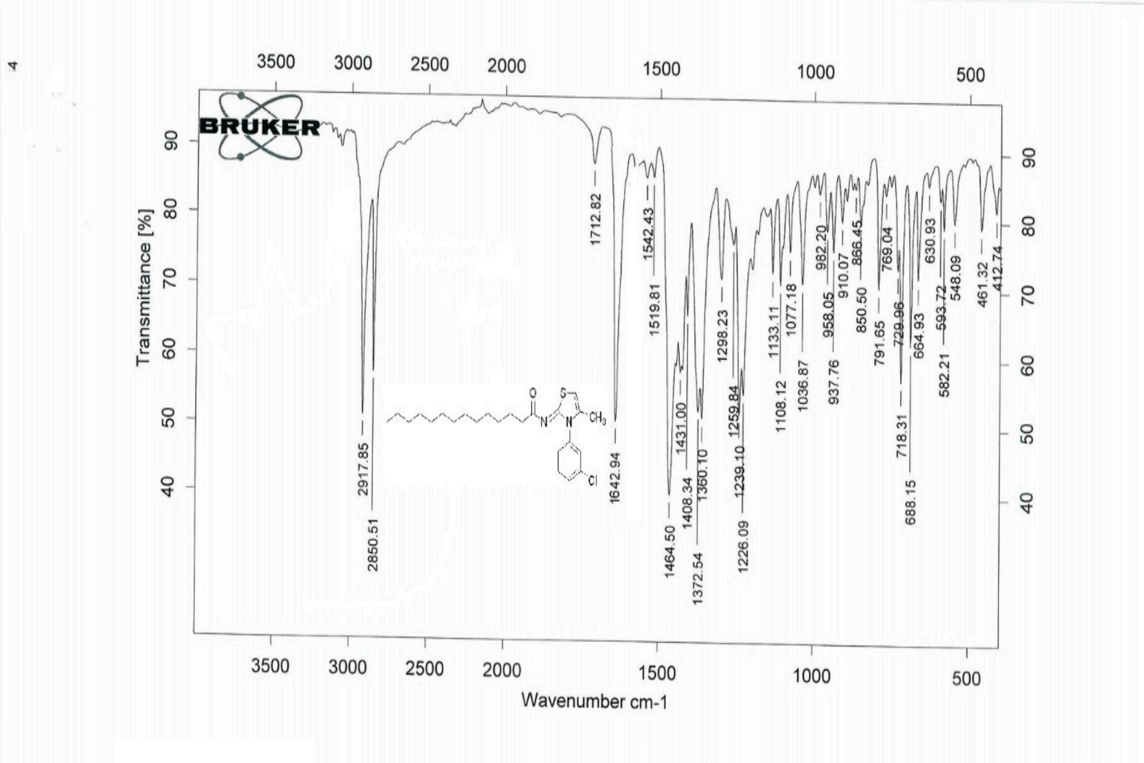

Figure S1. showing IR Spectra 2f.

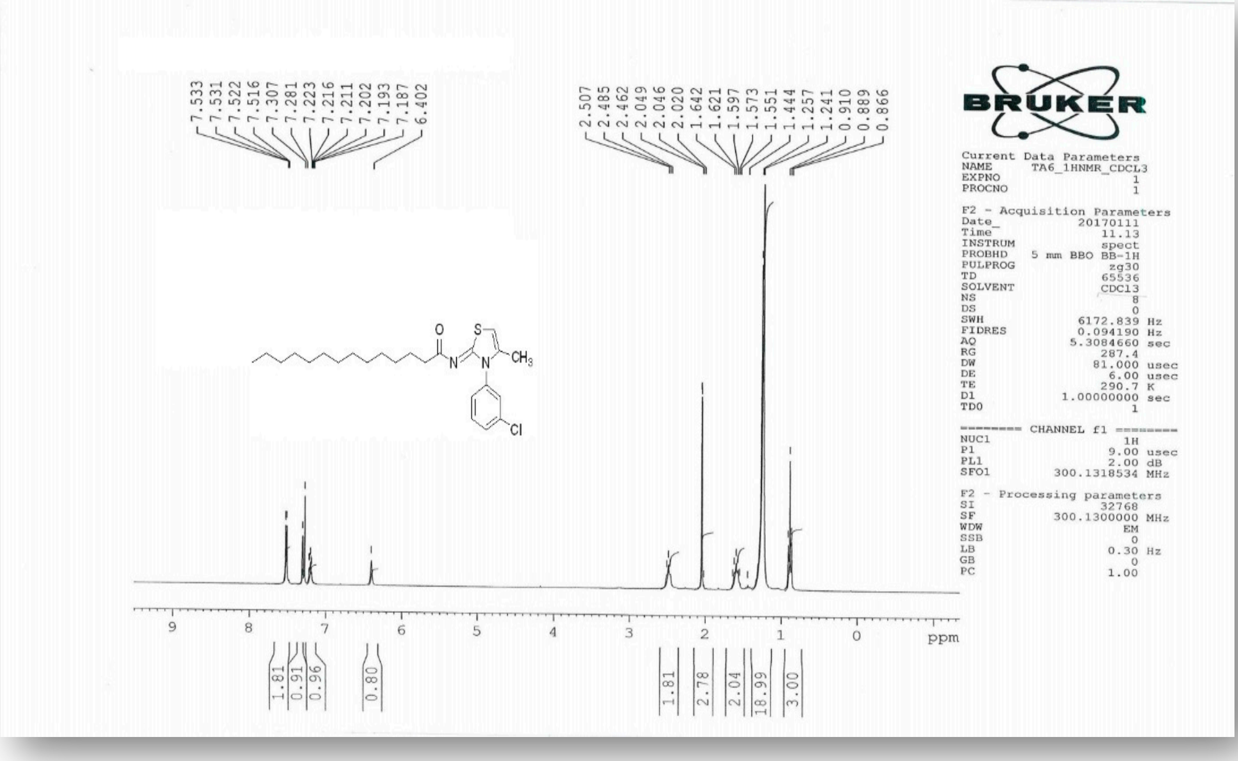

Figure S2. showing Proton NMR spectra 2f.

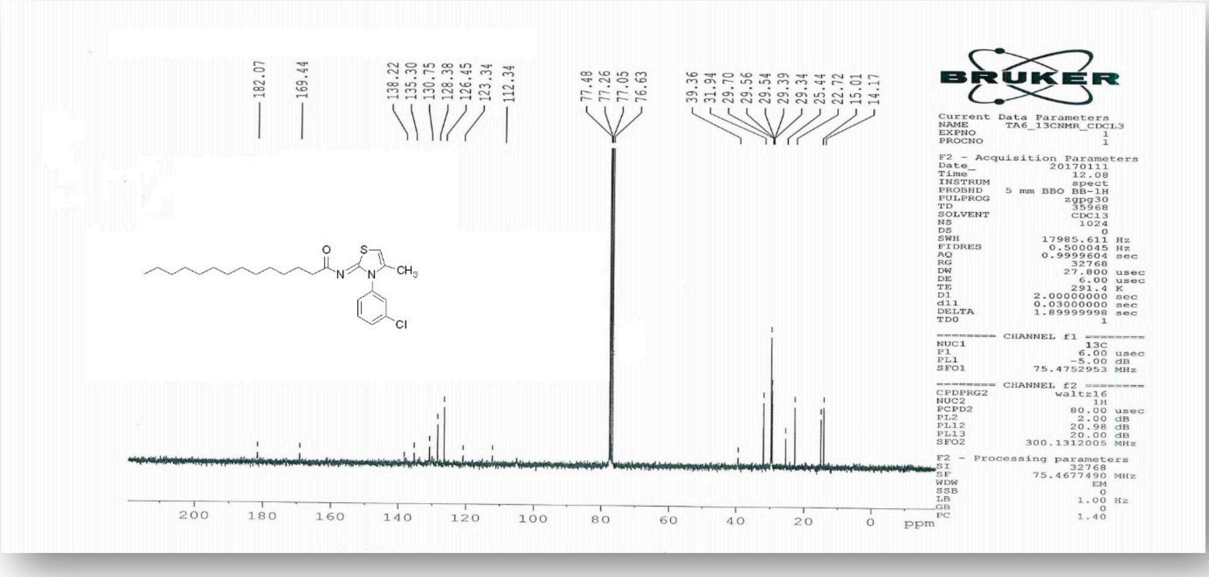

Figure S3. showing ^{13}C NMR spectra 2f.

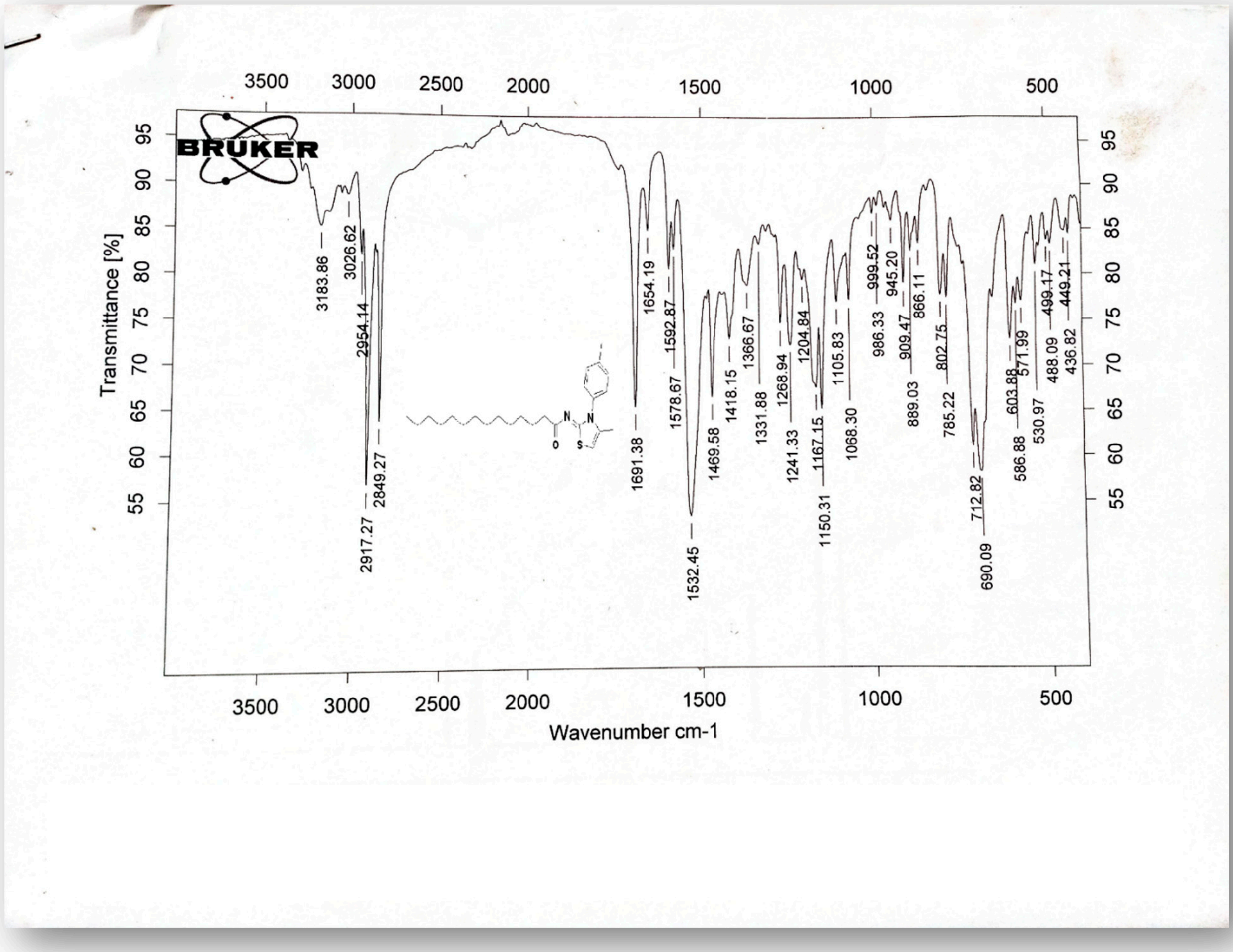

Figure S4. showing IR Spectra 2k.

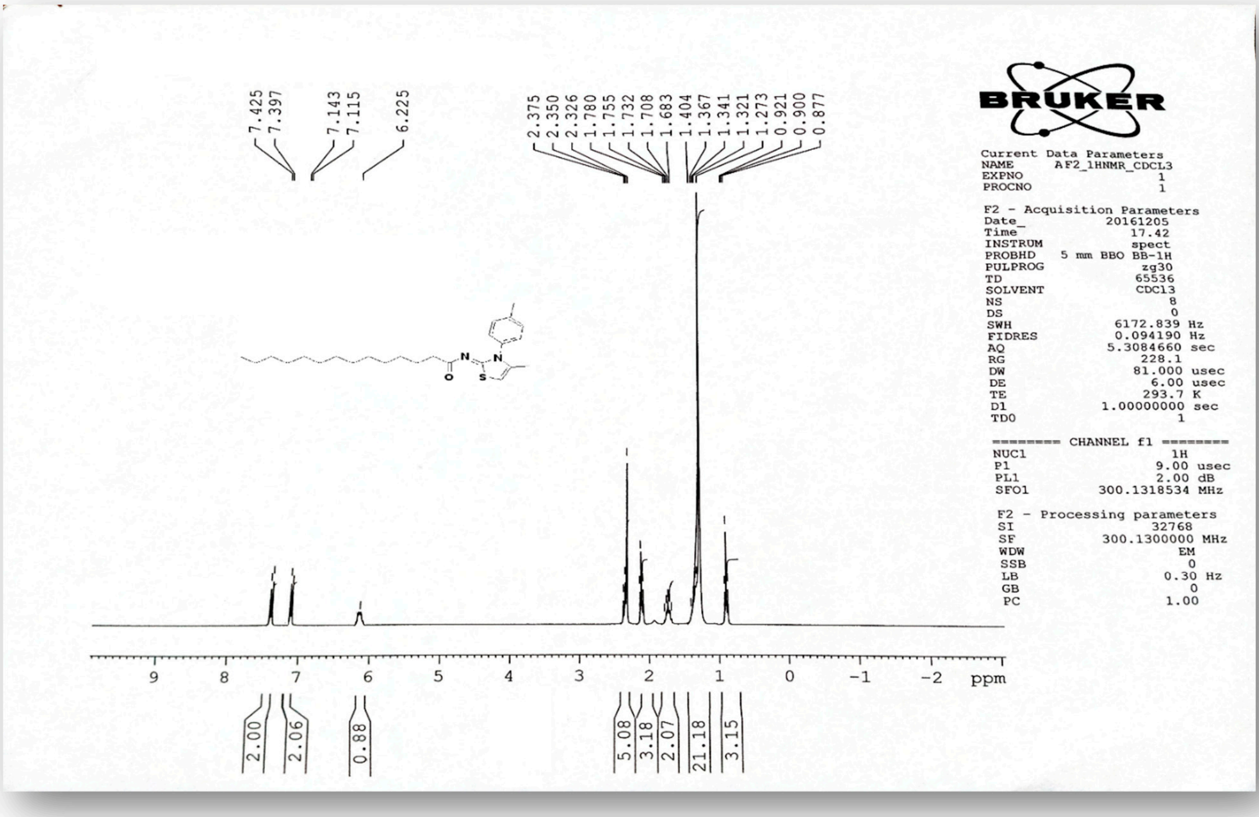

Figure S5. showing Proton NMR spectra 2k.

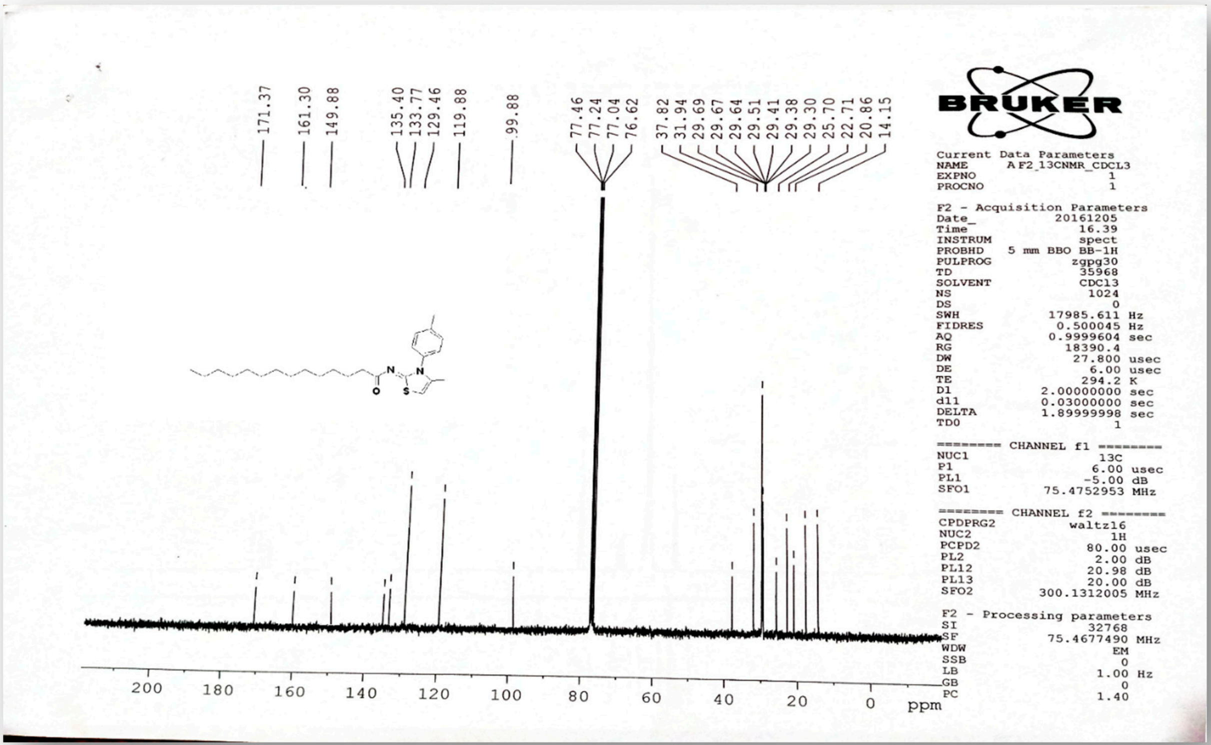

Figure S6. showing ^{13}C NMR spectra 2k.

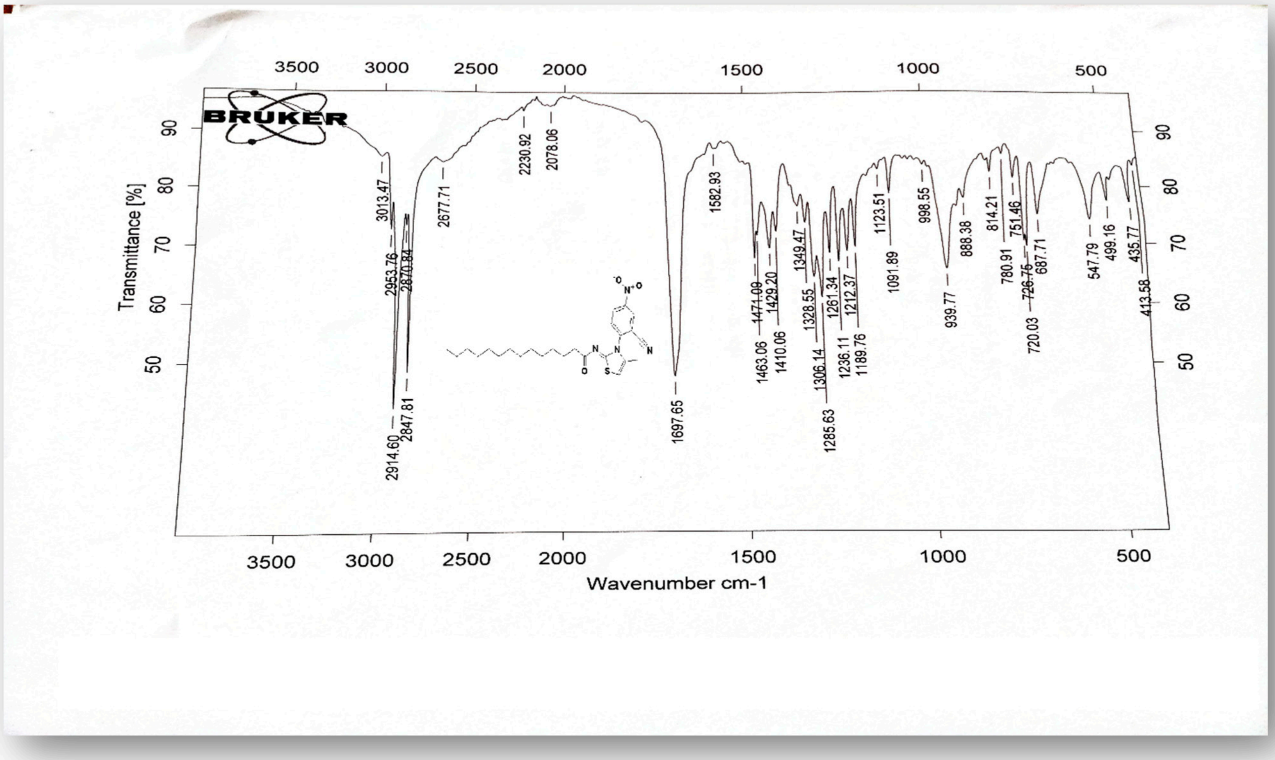

Figure S7. showing IR Spectra 2a.

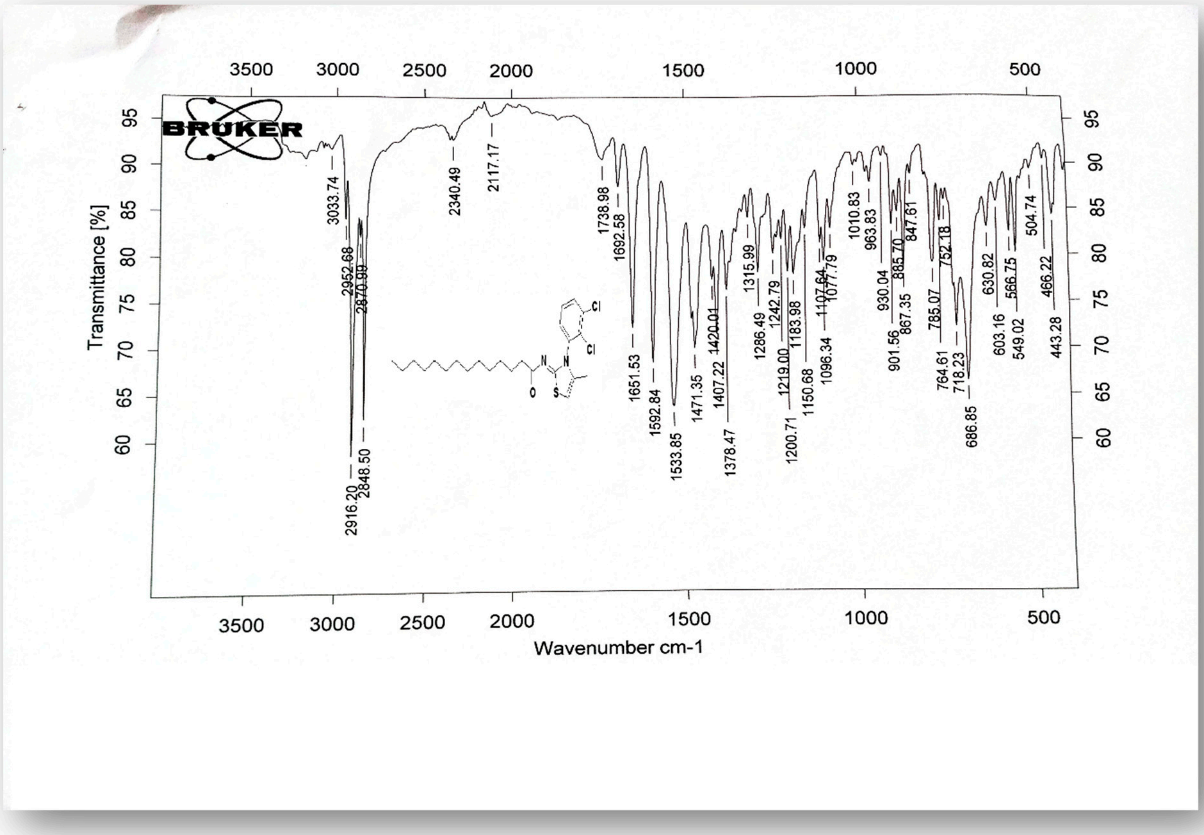

Figure S8. showing IR Spectra 2c.

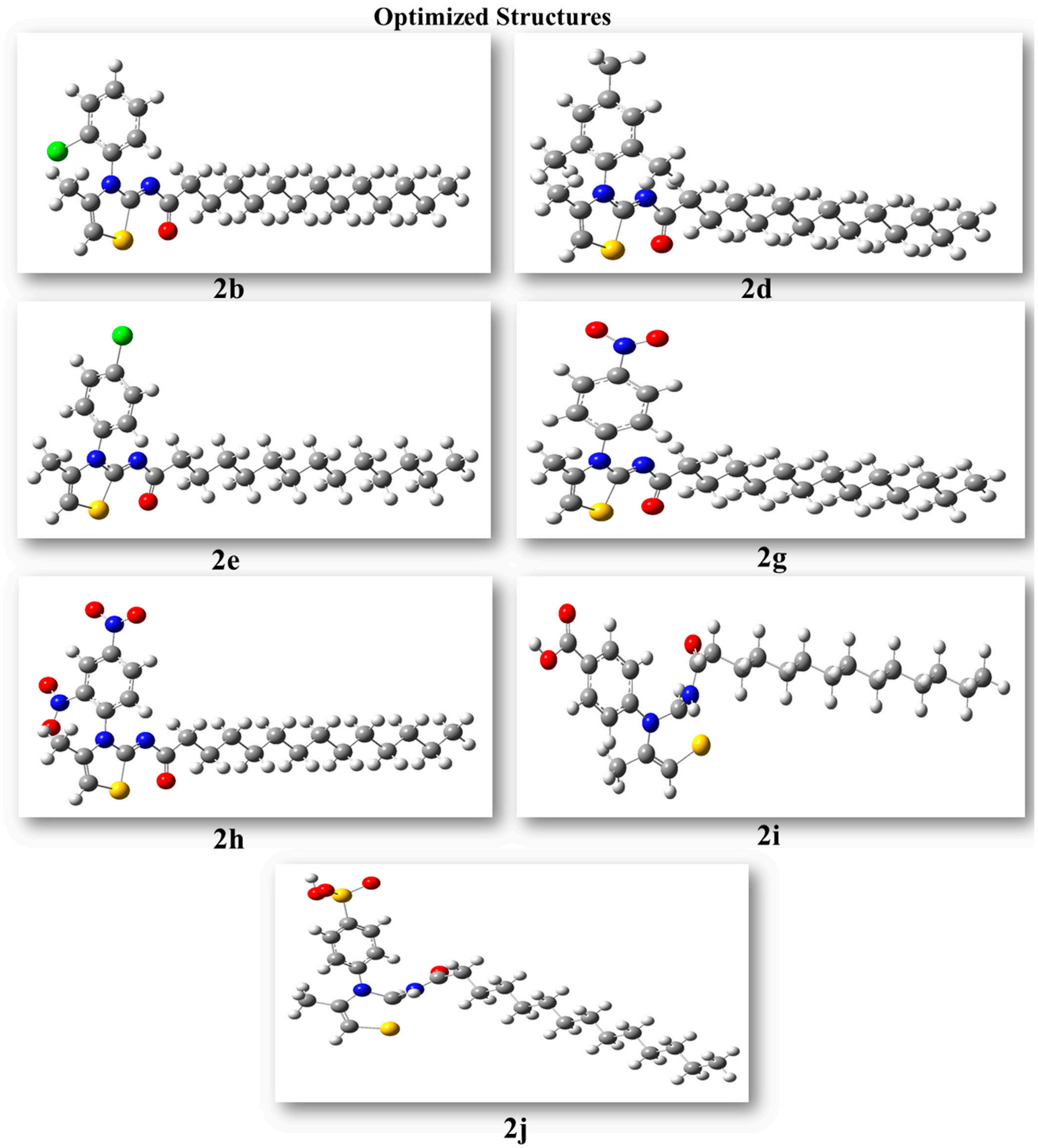

Figure S9. showing the optimized structures.

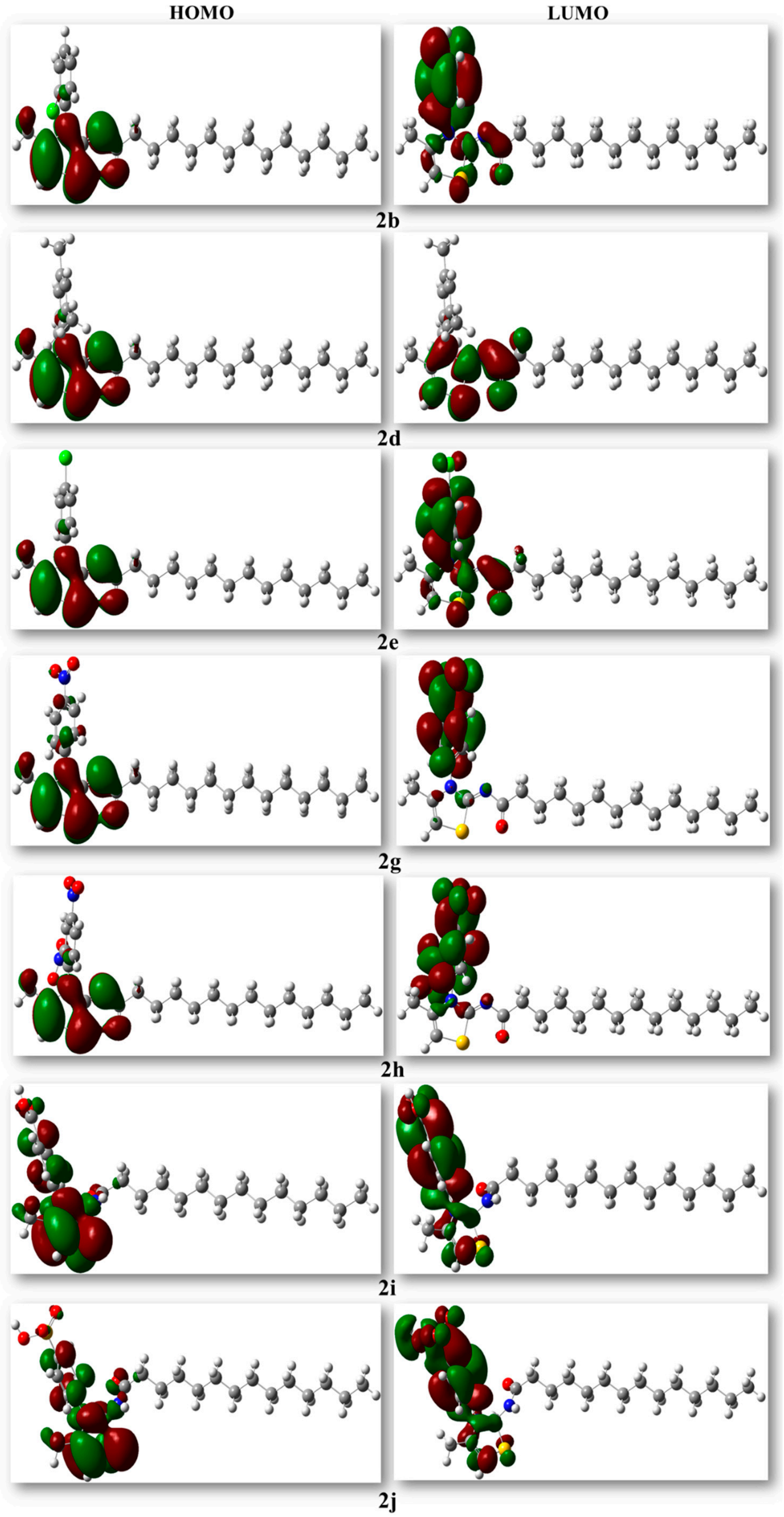

Figure S10. showing HOMO LUMO orbitals of synthesized derivatives.

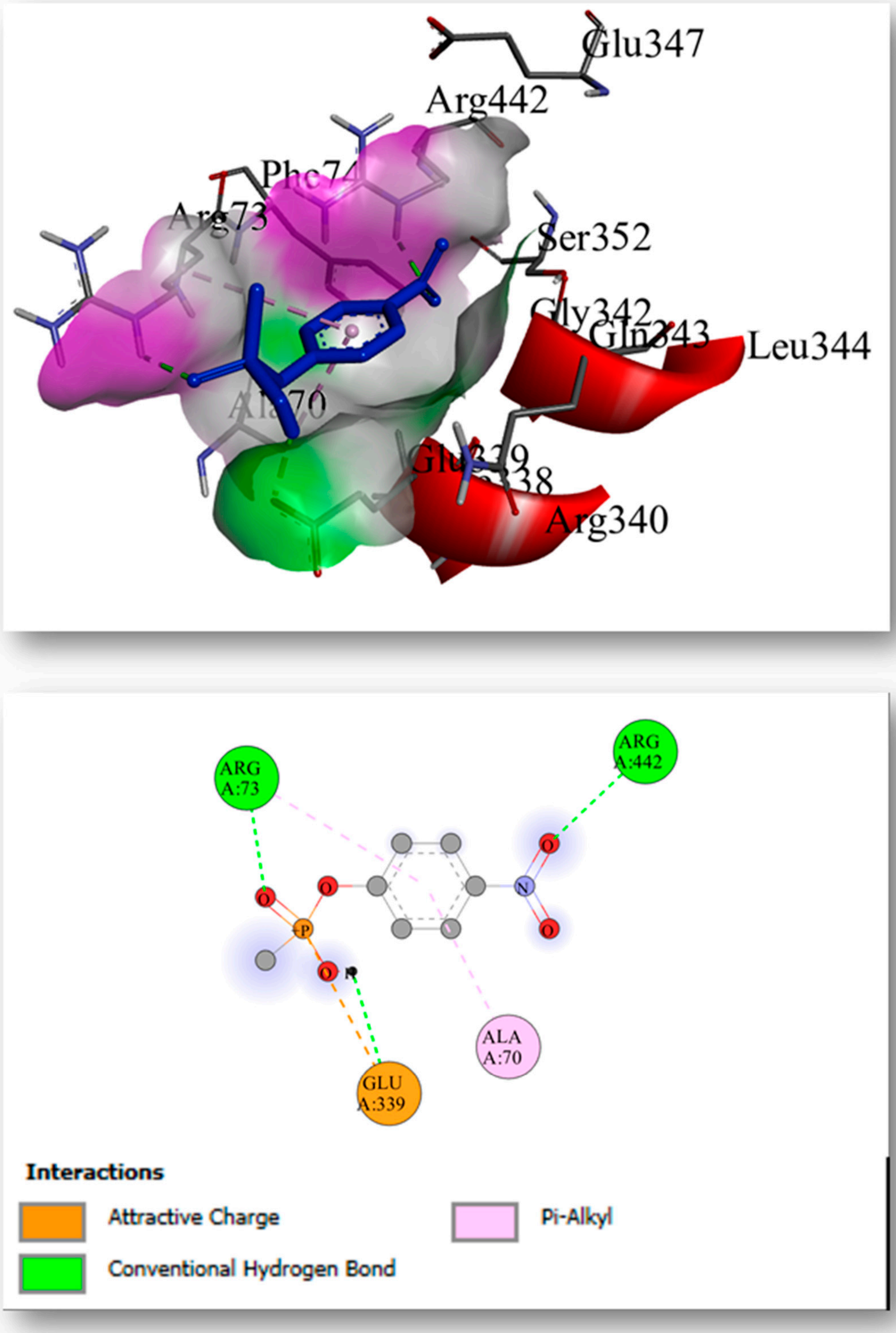

Figure S11. showing 2D and 3D interaction of Alkaline Phaosphatase with reference ligand Para Nitrophenyl Phosphonate.

Characterization Data of Synthesized Compound (2a-2k)

2-Tetradecanoylimino-3-(2-cyano-4-nitrophenyl)-4-methyl-1,3-thiazoline (2a)

Brown solid, Yield; 73%, m.p: 164oC, Rf = 0.39 (n-hexane: ethyl acetate, 1:1), FTIR υ (cm-1) 3013.4 (Sp^2 CH stretching) 2953.7 (Sp^3 CH stretching) 1697.6 (C=O), 1582.9 (Ar-C=C), 1471.0 (C=N), 1261.3 (C-S), 1189.7 (C-N); 1H NMR (DMSO-d6, 300 MHz,); δ (ppm) 8.19 (1H, d, Ar-H, J=6.5 Hz), 7.63 (d, 1H, Ar-H, J=3.9Hz), 7.43 (d, 1H, Ar-H, J= 4.1Hz), 6.48 (s, 1 H, CH=C), 2.31 (t, 2H, J=3.2Hz), 2.14 (quint, 2H), 2.05 (quint, 2H), 2.03 (quint, 2H),1.87 (quint, 2H), 1.81 (quint, 2H), 1.78 (quint, 2H), 1.63 (quint, 2H), 1.50 (quint, 2H), 1.42 (quint, 2H), 1.33 (quint, 2H), 1.26 (sex, 2H), 1.05 (s, 3H), 0.95 (t, 3H); 13C NMR (75 MHz DMSO-d6) δ (ppm) 177.3(C=O), 168.3, 138.6, 136.5, 134.6, 132.7, 131.3, 130.4 129.8, 118.3 (Ar-C), 117.3, 32.5, 31.2, 28.6, 27.4, 25.8, 23.7, 20.3, 19.8, 17.7, 16.3, 15.6, 13.2, 12.4, 11.3 Anal. Calcd. For C25H34N4O3S: C, 63.59; H, 7.38; N, 11.74; S, 6.71 found: C, 63.27; H, 7.17; N, 11.63; S, 5.50.

2-Tetradecanoylimino-3-(2-chlorophenyl)-4-methyl-1,3-thiazoline (2b)

Light blue solid, Yield; 76%, m.p: 163oC, Rf = 0.43 (n-hexane: ethyl acetate, 1:1), FTIR υ (cm-1) 1654.9 (C=O), 1572.4 (Ar-C=C), 1464.3 (C=N), 1249.1 (C-S), 1153.2 (C-N); 1H NMR (DMSO-d6, 300 MHz,); δ (ppm) 7.73 (1H, d, Ar-H, J=7.2 Hz), 7.63 (d, 1H, Ar-H, J=5.53Hz), 7.55 (d, 1H, Ar-H, J= 7.25Hz), 7.30 (d, 1H, Ar-H, J= 5.53Hz), 6.63 (s, 1 H, CH=C), 2.31 (t, 2H, J=3.1Hz), 2.28 (quint, 2H), 2.13 (quint, 2H), 2.02 (quint, 2H),1.89 (quint, 2H), 1.77 (quint, 2H), 1.69 (quint, 2H), 1.68(quint, 2H), 1.59 (quint, 2H), 1.46 (quint, 2H), 1.39 (quint, 2H), 1.32 (sex, 2H), 1.19 (s, 3H), 0.98 (t, 3H); 13C NMR (75 MHz DMSO-d6) δ (ppm) 176.7(C=O), 164.3, 137.5, 136.5, 135.4, 133.5, 131.3, 130.7, 128.3, 118.8 (Ar-C), 34.6, 32.1, 27.6, 25.4, 23.7, 21.8, 19.7, 18.1, 16.7, 15.1, 14.6, 13.9, 12.4, 11.8 Anal. Calcd. For C24H35ClN2OS: C, 66.02; H, 7.98; N, 6.34; S, 7.26 found: C, 65.93; H, 7.72; N, 6.23; S, 7.14

2-Tetradecanoylimino-3-(2,3-dichlorophenyl)-4-methyl-1,3-thiazoline (2c)

Light yellow crystalline solid, Yield; 71%, m.p: 195oC, Rf = 0.45 (n-hexane: ethyl acetate, 1:1), FTIR υ (cm-1) 3033.7 (Sp^2 CH stretching) 2916.2 (Sp^3 CH stretching) 1692 (C=O), 1592 (Ar-C=C), 1471 (C=N), 1286 (C-S), 1183 (C-N); 1H NMR (DMSO-d6, 300 MHz,); δ (ppm) 7.31-6.26 (3H, m, Ar-H), 6.73 (s, 1 H, CH=C), 2.25 (t, 2H, J=3Hz), 2.16 (quint, 2H), 2.10 (quint, 2H), 1.96 (quint, 2H),1.87 (quint, 2H), 1.75 (quint, 2H), 1.64 (quint, 2H), 1.59(quint, 2H), 1.54 (quint, 2H), 1.48 (quint, 2H), 1.38 (quint, 2H), 1.27 (sex, 2H), 1.16 (s, 3H), 1.08 (t, 3H); 13C NMR (75 MHz DMSO-d6) δ (ppm) 175.7(C=O), 167.5, 138.5, 137.5, 136.6, 135.8, 134.6, 129.7, 126.6, 117.9 (Ar-C), 34.6, 33.6, 29.3, 26.6, 24.7, 23.8, 20.4, 19.5, 18.4, 17.1, 15.3, 13.6, 12.4, 11.8 Anal. Calcd. For C24H34Cl2N2OS: C, 61.30; H, 7.15; N, 5.86; S, 6.73 found: C, 61.20; H, 7.07; N, 5.63; S, 6.54.

2-Tetradecanoylimino-3- mesityl -4-methyl-1,3-thiazoline (2d)

Black solid, Yield; 77%, m.p: 174oC, Rf = 0.36 (n-hexane: ethyl acetate, 1:1), FTIR υ (cm-1) 1645.6 (C=O), 1562.7 (Ar-C=C), 1434.5 (C=N), 1269.7 (C-S), 1143.2 (C-N); 1H NMR (DMSO-d6, 300MHz,); δ (ppm) 7.53 (2H, s, Ar-H), 6.68 (s, 1 H, CH=C), 3.20 (s, 9H, -CH3), 2.32 (t, 2H, J=2Hz), 2.28 (quint, 2H), 2.16 (quint, 2H), 1.97 (quint, 2H), 1.81 (quint, 2H), 1.76 (quint, 2H), 1.67 (quint, 2H), 1.61(quint, 2H), 1.57 (quint, 2H), 1.44 (quint, 2H), 1.32 (quint, 2H), 1.21 (sex, 2H), 1.19 (s, 3H), 1.13 (t, 3H); 13C NMR (75 MHz DMSO-d6) δ (ppm) 176.7(C=O),

169.6, 138.6, 138.3, 135.6, 133.3, 130.6, 128.7, 128.2, 117.3 (Ar-C), 35.9, 34.6, 31.3, 29.9, 27.7, 26.7, 24.2, 22.5, 20.7, 18.4, 17.8, 16.4, 15.4, 14.6, 13.3, 12.4, 11.3 Anal. Calcd. For C27H42N2OS: C, 73.12; H, 9.35; N, 6.21; S, 7.15 found: C, 72.97; H, 9.24; N, 6.13; S, 7.02.

2-Tetradecanoylimino-3-(4-chlorophenyl)-4-methyl-1,3-thiazoline (2e)

Light brown solid, Yield; 81%, m.p: 187oC, Rf = 0.41 (n-hexane: ethyl acetate, 1:1), FTIR υ (cm-1) 1646.9 (C=O), 1569.4 (Ar-C=C), 1484.5 (C=N), 1253.1 (C-S), 1154.2 (C-N); 1H NMR (DMSO-d6, 300 MHz,); δ (ppm) 7.76 (d, 2H, Ar-H, J=8.35 Hz), 7.43 (d, 2H, Ar-H, J=8.03Hz), 6.83 (s, 1 H, CH=C), 2.21 (t, 2H, J=3.0Hz), 2.19 (quint, 2H), 2.17 (quint, 2H), 2.08 (quint, 2H), 1.97 (quint, 2H), 1.85 (quint, 2H), 1.79 (quint, 2H), 1.67 (quint, 2H), 1.61 (quint, 2H), 1.45 (quint, 2H), 1.36 (quint, 2H), 1.23 (sex, 2H), 1.13 (s, 3H), 1.09 (t, 3H); 13C NMR (75 MHz DMSO-d6) δ (ppm) 177.7(C=O), 166.8, 136.5, 135.5, 133.4, 131.9, 131.3, 128.7, 128.2, 117.2 (Ar-C), 34.6, 32.1, 29.6, 27.4, 25.7, 23.8, 21.7, 19.1, 17.7, 16.9, 14.6, 13.7, 12.8, 11.6 Anal. Calcd. For C24H35ClN2OS: C, 66.16; H, 8.01; N, 6.34; S, 7.21 found: C, 66.05; H, 7.91; N, 6.23; S, 7.10.

2-Tetradecanoylimino-3-(3-chlorophenyl)-4-methyl-1,3-thiazoline (2f)

Light yellow solid, Yield; 85%, m.p: 195oC, Rf = 0.34 (n-hexane: ethyl acetate, 1:1), FTIR υ (cm-1) 3020 at C-H aromatic 2917 at C-H thiazoline, 1642.9 (C=O), 1542.4 (Ar-C=C), 1464.5 (C=N), 1239.1 (C-S), 1133.2 (C-N); 1H NMR ($CDCl_3$, 300 MHz,); δ (ppm) 7.53 (dd, 1H, J = 7.5, 7.2 Ar-H), 7.51 (d, 1H, J =7.5, Ar-H), 7.30 (d, 1H, J = 7.1, Ar-H), 7.21 (d, 1H, J =7.2, Ar-H), 6.40 (s, 1 H, CH=C), 2.50 (t, 2H, J=3.0Hz), 2.40 (quint, 2H), 2.17 (quint, 2H), 2.09 (quint, 2H),1.95 (quint, 2H), 1.83 (quint, 2H), 1.75 (quint, 2H), 1.63 (quint, 2H), 1.54 (quint, 2H), 1.51 (quint, 2H), 1.41 (quint, 2H), 1.11 (sex, 2H), 0.88 (s, 3H), 0.86 (t, 3H) ; 13C NMR (75 MHz $CDCl_3$) δ (ppm) 182.1(C=O), 169.4, 138.1, 135.71, 130.3, 128, 126.5, 124.3, 123.4, 112.4, (Ar-C), 39.4, 39.1, 29.6, 27.3, 25.6, 24.7, 23.8, 21.5, 19.7, 18.3, 17.6, 16.9, 15.0, 14.2, Anal. Calcd. For C24H35ClN2OS: C, 66.13; H, 8.01; N, 6.31; S, 7.11 found: C, 65.97; H, 7.91; N, 6.23; S, 6.95.

2-Tetradecanoylimino-3-(4-nitrophenyl)-4-methyl-1,3-thiazoline (2g)

Black solid, Yield; 79%, m.p: 174oC, Rf = 0.34 (n-hexane: ethyl acetate, 1:1), FTIR υ (cm-1) 1682.6 (C=O), 1553.3 (Ar-C=C), 1463.7 (C=N), 1236.3 (C-S), 1136.9 (C-N); 1H NMR (DMSO-d6, 300 MHz,); δ (ppm) 7.65 (d, 2H, Ar-H, J=8.01 Hz), 7.43 (d, 2H, Ar-H, J=7.43Hz), 6.38 (s, 1 H, CH=C), 2.23 (t, 2H, J=3Hz), 2.16 (quint, 2H), 2.13 (quint, 2H), 2.08 (quint, 2H),1.94 (quint, 2H), 1.84 (quint, 2H), 1.71 (quint, 2H), 1.63 (quint, 2H), 1.58 (quint, 2H), 1.44 (quint, 2H), 1.35 (quint, 2H), 1.28 (sex, 2H), 1.16 (s, 3H), 1.08 (t, 3H); 13C NMR (75 MHz DMSO-d6) δ (ppm) 176.7(C=O), 168.3, 138.5, 137.5, 135.8, 133.4, 132.2, 130.8, 129.2, 118.1 (Ar-C), 35.6, 34.1, 32.4, 29.4, 28.7, 25.8, 23.7, 20.5, 18.7, 17.9, 15.5, 14.6, 12.3, 11.9 Anal. Calcd. For C24H35N3O3S: C, 64.53; H, 7.76; N, 9.31; S, 7.11 found: C, 64.37; H, 7.65; N, 9.23; S, 7.01.

2-Tetradecanoylimino-3-(2,4-dinitrophenyl)-4-methyl-1,3-thiazoline (2h)

Dark brown solid, Yield; 76%, m.p: 165oC, Rf = 0.37 (n-hexane: ethyl acetate, 1:1), FTIR υ (cm-1) 1647.2 (C=O), 1537.8 (Ar-C=C), 1473.9 (C=N), 1272.1 (C-S), 1173.6 (C-N); 1H NMR (DMSO-d6, 300 MHz,); δ (ppm) 8.63 (1H, d, Ar-H, J=6.8 Hz), 8.32 (d, 1H, Ar-H, J=4.5Hz), 7.63 (d, 1H, Ar-H, J= 4.2Hz), 6.37 (s, 1 H, CH=C), 2.36 (t, 2H, J=3Hz), 2.30 (quint, 2H), 2.16 (quint, 2H), 2.03 (quint, 2H),1.95 (quint, 2H), 1.62 (quint, 2H), 1.57 (quint, 2H), 1.52 (quint,

2H), 1.45 (quint, 2H), 1.38 (quint, 2H), 1.31 (quint, 2H), 1.23 (sex, 2H), 1.13 (s, 3H), 1.06 (t, 3H); 13C NMR (75 MHz DMSO-d6) δ (ppm) 176.3 (C=O), 168.4, 138.9, 136.5, 135.3, 133.7, 132.6, 131.4, 127.9, 117.4 (Ar-C), 35.5, 33.2, 29.7, 27.8, 25.9, 24.7, 21.3, 19.7, 18.3, 16.9, 14.6, 12.7, 11.9, 11.6 Anal. Calcd. For C24H34N4O5S: C, 58.59; H, 6.83; N, 11.24; S, 6.34 found: C, 58.47; H, 6.67; N, 11.03; S, 6.24.

4-(4-Methyl-2-tetradecanamidothiazol-3(2H)-yl)benzoic acid (2i)

Light brown solid, Yield; 80%, m.p: 164oC, Rf = 0.33 (n-hexane: ethyl acetate, 1:1), FTIR υ (cm-1) 1672.8 (C=O), 1553.7 (Ar-C=C), 1457.5 (C=N), 1237.7 (C-S), 1154.2 (C-N); 1H NMR (DMSO-d6, 300 MHz,); δ (ppm) 11.89 (s, 1H, COOH), 7.85 (d, 2H, Ar-H, J=8.25 Hz), 7.43 (d, 2H, Ar-H, J=7.26Hz), 6.52 (s, 1 H, CH=C), 2.23 (t, 2H, J=3.0Hz), 2.12 (quint, 2H), 2.08 (quint, 2H), 1.98 (quint, 2H),1.86 (quint, 2H), 1.74 (quint, 2H), 1.69 (quint, 2H), 1.64 (quint, 2H), 1.57 (quint, 2H), 1.44 (quint, 2H), 1.32 (quint, 2H), 1.25 (sex, 2H), 1.15 (s, 3H), 1.09 (t, 3H); 13C NMR (75 MHz DMSO-d6) δ (ppm) 178.7(C=O), 172.3, 169.7, 138.5, 137.5, 135.9, 134.6, 133.1, 129.7, 129.1, 117.7 (Ar-C), 33.6, 32.1, 29.5, 26.4, 25.7, 24.8, 21.7, 19.1, 17.7, 15.9, 14.6, 13.1, 12.8, 11.3 Anal. Calcd. For C25H36N2O3S: C, 67.43; H, 8.06; N, 6.20; S, 7.11 found: C, 67.33; H, 7.91; N, 6.13; S, 7.01.

4-(4-Methyl-2-tetradecanamidothiazol-3(2H)-yl)benzenesulfonic acid (2j)

Black solid, Yield; 87%, m.p: 166oC, Rf = 0.43 (n-hexane: ethyl acetate, 1:1), FTIR υ (cm-1) 1682.1 (C=O), 1573.6 (Ar-C=C), 1434.8 (C=N), 1242.6 (C-S), 1163.4 (C-N); 1H NMR (DMSO-d6, 300 MHz,); δ (ppm) 7.76 (d, 2H, Ar-H, J=7.52 Hz), 7.58 (d, 2H, Ar-H, J=7.25Hz), 6.64 (s, 1 H, CH=C), 3.01 (s, 1H, SO3H), 2.22 (t, 2H, J=2.5Hz), 2.19 (quint, 2H), 2.10 (quint, 2H), 1.89 (quint, 2H),1.74 (quint, 2H), 1.65 (quint, 2H), 1.57 (quint, 2H), 1.52 (quint, 2H), 1.46 (quint, 2H), 1.41 (quint, 2H), 1.32 (quint, 2H), 1.24 (sex, 2H), 1.18 (s, 3H), 1.07 (t, 3H); 13C NMR (75 MHz DMSO-d6) δ (ppm) 177.8(C=O), 169.4, 138.5, 137.9, 136.6, 135.4, 133.8, 130.7, 129.1 117.6 (Ar-C), 34.6, 33.4, 31.6, 28.4, 26.7, 24.8, 22.7, 20.1, 18.7, 16.9, 14.6, 13.1, 12.6, 11.9, Anal. Calcd. For C24H36N2O4S2: C, 59.79; H, 7.45; N, 5.72; S, 13.21 found: C, 59.57; H, 7.31; N, 5.63; S, 13.10.

2-Tetradecanoylimino-3-p-tolyl-4-methyl-1,3-thiazoline (2k)

Yellow solid, Yield; 75%, m.p: 146oC, Rf = 0.45 (n-hexane: ethyl acetate, 1:1), FTIR υ (cm-1) 3026 (Sp^2 CH stretching) 2917 (Sp^3 CH stretching) 1691 (C=O), 1532 (Ar-C=C), 1418 (C=N), 1241 (C-S), 1167 (C-N); 1H NMR ($CDCl_3$ 300 MHz,); δ (ppm) 7.42 (d, 2H, Ar-H, J=7.5 Hz), 7.14 (d, 2H, Ar-H, J=7.2Hz), 6.22 (s, 1 H, CH=C), 2.37 (s, 1H, CH3), 2.35 (t, 2H, J=3Hz), 2.32 (quint, 2H), 1.78 (quint, 2H), 1.75 (quint, 2H),1.73 (quint, 2H), 1.70 (quint, 2H), 1.68 (quint, 2H), 1.40 (quint, 2H), 1.36 (quint, 2H), 1.34 (quint, 2H), 1.32 (quint, 2H), 1.27 (sex, 2H), 0.92 (s, 3H), 0.87 (t, 3H); 13C NMR (75 MHz $CDCl_3$) δ (ppm) 171.3(C=O), 161.1, 149.8, 135.4, 133.7, 129.4, 119.8, 99.8, (Ar-C), 37.8, 31.9, 29.6, 29.4, 29.3, 25.7, 22.7, 20.8, 14.1, Anal. Calcd. For C25H38N2OS: C, 72.32; H, 9.14; N, 6.65; S, 7.53 found: C, 72.23; H, 9.03; N, 6.53; S, 7.33.
